# Assessment of different techniques for 3D superimposition of serial digital maxillary dental casts on palatal structures

**DOI:** 10.1038/s41598-017-06013-5

**Published:** 2017-07-19

**Authors:** Georgios Vasilakos, Roman Schilling, Demetrios Halazonetis, Nikolaos Gkantidis

**Affiliations:** 1 Department of Orthodontics and Dentofacial Orthopedics, Friedrich Schiller University of Jena, An der alten Post 4, 07743 Jena, Germany; 2 Department of Orthodontics and Dentofacial Orthopedics, University of Bern, Freiburgstrasse 7, CH-3010 Bern, Switzerland; 3 Department of Orthodontics, School of Dentistry, National and Kapodistrian University of Athens, 2 Thivon Street, Goudi, 11527 Athens, Greece; 4 Department of Orthodontics and Dentofacial Orthopedics, University of Bern, Freiburgstrasse 7, CH-3010 Bern, Switzerland

## Abstract

Serial 3-dimensional dental model superimposition provides a risk-free, detailed evaluation of morphological alterations on a patient’s mouth. Here, we evaluated accuracy and precision of five palatal areas, used for superimposition of maxillary 3D digital dental casts. Sixteen pre- and post-orthodontic treatment dental casts of growing patients (median time lapse: 15.1 months) were superimposed on each palatal area using the iterative closest point algorithm. Area A (medial 2/3 of the third rugae and a small area dorsal to them) was considered the gold standard, due to high anatomical stability. Areas B, C, and D added a distal extension along the midpalatal raphe, an anterior extension to the second rugae, and the remaining palatal surface, respectively. Area E was similar to A, located more posteriorly. Non parametric multivariate models showed minimal or no effect on accuracy and precision by operator, time point, or software settings. However, the choice of superimposition area resulted in statistically significant differences in accuracy and clinically significant differences in detected tooth movement (95% limits of agreement exceeding 1 mm and 3°). Superimposition on area A provided accurate, reproducible, and precise results. Outcomes were comparable for area B, but deteriorated when alternative areas were used.

## Introduction

The use of 3 dimensional (3D) digital dental casts has widely expanded in recent years. Important advantages of digital over conventional stone casts include no need for physical storage, risk- and cost-free transfer and extensive potential for data processing. Even when there is need for physical dental models, they can still be printed by 3D printers. The accuracy of digital models has been thoroughly tested so far and is considered adequate^1^.

The superimposition of serial images of the same patient is of great importance both for clinical and research purposes^2^. Superimposition of dental models is highly preferable compared to radiographically obtained models, since impression taking or direct 3D intraoral scanning is a risk-free procedure with no radiation concerns. In order to assess changes in an area of interest, a stable reference area or stable reference points are sought to successfully register the two (or more) serial images.

Various techniques have been proposed so far for superimposing 3D digital casts^3–5^. Two studies in non-growing patients that used miniscrews as a gold standard superimposition reference suggested the medial part of the third rugae and a small region dorsal to it as a stable superimposition reference^3, 4^. The third rugae have also been suggested as the most stable rugae by other previous studies^6, 7^. Studies on non-growing patients that compared tooth movement in cephalometric radiographs with that measured on superimposed digital models suggested a mushroom-shaped palatal area including the rugae as a head^8^ or the placement of landmarks on the third ruga^9^. The use of three points in the incisive papilla area was also suggested^5^. However, all the above studies tested differences in mean values and did not test for differences between individual measurements, as required for such hypotheses^10, 11^.

In growing subjects, growth is expected to occur in the palate including the rugae region^4, 12–16^. Palatal depth and surface area also increase during growth^13, 17^, as do the dentoalveolar processes, especially in the transitional period of the development of the dentition^18, 19^. Thus, superimposition of dental casts in growing subjects is expected to be even more challenging. So far only one study has tested the validity of tooth movement assessment in superimposed dental models of growing patients^20^, using almost the whole palate as the superimposition reference area. The measurements were compared to findings from lateral cephalometric radiographs and showed very high correlations in the anteroposterior level. However, Bland-Altman plots revealed that differences between the two techniques were relatively large. Unfortunately, potential reasons for this were not tested.

Therefore, the aim of the present study was to assess the efficacy of alternate superimposition reference areas and suggest a simple, efficient, and accurate way to detect, visualize, and quantify tooth movement in growing patients through superimposing serial 3D dental models. For this purpose, we assessed five different superimposition techniques, on four previously used palatal areas and one used for first time. The superimposition on the medial 2/3 of the third rugae and a small area dorsal to them, was considered the gold standard technique, because of its higher anatomical form stability^3, 4, 6, 7, 9, 12^.

## Methods

### Sample

The material for this study consisted of existing pre-treatment (T0) and follow-up (T1) maxillary dental casts of 16 growing patients (7 males and 9 females; median age at T0: 8.0, range: 6.0–9.3 years) that were all at the early or transitional mixed dentition stage at T0. The follow-up model was obtained at least 6 months after the end of active treatment. The median time lapse between two serial models was 15.1 months (range: 7.2–21.8). All patients were treated for dental anterior crossbite (at least one maxillary incisor) through the placement of resin modified glass ionomer cement (Ultra Band-Lok Blue, Reliance Orthodontic Products, 1540 West Thorndale Ave, Itasca, IL 60143) at the occlusal surfaces of selected lower posterior teeth^21^, in a private practice in Cologne (Germany). From the variety of dental casts available in the practice, this sample was selected because this minimally invasive approach leads to quantifiable tooth movement of specific teeth in growing patients, while it does not directly affect palatal morphology. Witnessed written informed consents were obtained from all patients and their parents for the use of their data.

The dental casts were obtained according to the regular protocol of the practice, through alginate impressions (Tetrachrom Alginat, KANIEDENTA GmbH & Co. KG, Zum Haberland 36, D-32051 Herford, Germany), which were poured with plaster (Alabaster Klasse 3, Wiegelmann Dental GmbH, Landsberger Strasse 6, D 53119 Bonn, Germany) within the day when the impression was taken. Each cast was scanned with a 3D surface scanner (stripe light/LED illumination; accuracy <20 μm; Laboratory scanner D104a, Cendres + Métaux SA, Rue de Boujean 122, CH-2501 Biel/Bienne) to obtain the 3D STL models used in the study. Each maxillary 3D mesh consisted of approximately 325.000 vertices.

### Superimposition procedure

Five different superimposition techniques on five different palatal areas were tested. All superimpositions were performed using Viewbox 4 software (version 4.1.0.1 BETA, dHAL Software, Kifissia, Greece) as described below.

#### Superimposition reference areas and the teeth of interest

**Figure 1:**
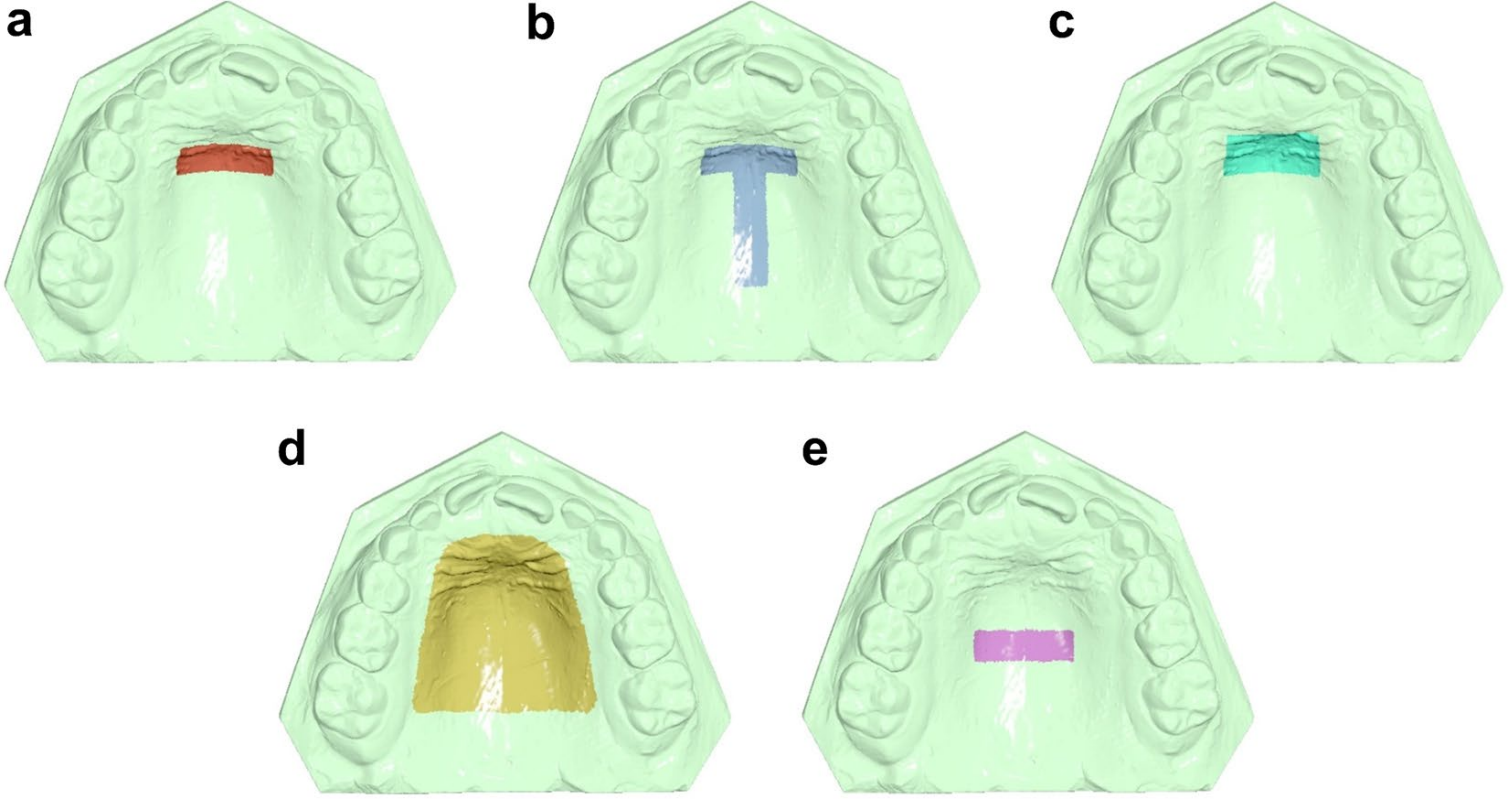
Superimposition reference areas used in the study. (**a**) The area of the palate limited anteriorly by the medial 2/3 of the third rugae and laterally by two lines parallel to the midpalatal suture and extending posteriorly 5 mm from the third rugae. (**b**) Area A, plus a 6 mm wide stripe on the midpalatal suture extending posteriorly to the level of a line connecting the lingual grooves of the 1^st^ permanent molars at the gingival level. (**c**) An area similar to A, but starting anteriorly from the medial 2/3 of the second rugae. (**d**) Almost the whole palate delimited by a line 5 mm distant from all gingival margins and extending posteriorly until a line connecting the lingual grooves of the 1^st^ permanent molars at the gingival level. (**e**) An area of similar extent and transversal position to area A, but starting anteriorly at a line that connects the interproximal areas of the primary molars.

After importing the T0 and T1 STL files in Viewbox 4 software, five superimposition reference areas were selected on the T0 model: A) a small area of the palate including the medial 2/3 of the third rugae and the area 5 mm dorsal to them (Fig. 1a)^3^, B) area A, plus a 6 mm wide stripe on the midpalatal suture extending posteriorly to the level of a line connecting the lingual grooves of the 1^st^ permanent molars (Fig. 1b)^8^, C) an area similar to area A, but starting anteriorly from the medial 2/3 of the second rugae (Fig. 1c)^8^, D) almost the whole palate delimited by a line 5 mm distant from all gingival margins and extending posteriorly until the middle of the 1st permanent molars (Fig. 1d)^20^, and E) an area of similar extent and transversal position to area A, but starting anteriorly at a line which connects the interproximal areas between the primary molars of each side (Fig. 1e).

Superimposing on area A was considered the gold standard technique in this study. Maximum congruence of the two models is expected in the specific area due to its anatomical form stability^3, 4, 6, 7, 9, 12^, which was also not directly affected by treatment.

Furthermore, in the T0 model, the clinical crown of one central incisor (the mostly erupted) and the right and left second primary molars were selected as the teeth where movement from T0 to T1 was to be assessed.

#### Implementation of the superimpositions

The T0 and T1 3D models of each patient were superimposed, in each reference area described above, using the software’s implementation of the iterative closest point algorithm (ICP)^22^ at two different settings. The basic setting (1) was: 100% estimated overlap of meshes, matching point to plane, exact nearest neighbor search, 100% point sampling, 50 iterations, and the alternative setting (2) was: same as (1), but with 98% estimated overlap of meshes.

**Figure 2:**
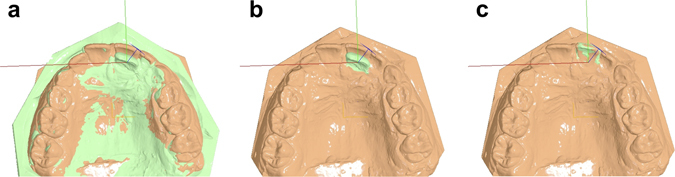
Example of superimposition and tooth movement assessment. (**a**) Superimposed pre-treatment (green) and follow-up (peach) models on area A. (**b**) The tooth of interest (left central incisor) was selected on the pre-treatment model (green). The axes used for the assessment of positional changes of the tooth of interest originated from the centroid of this tooth in the pre-treatment position and were oriented according to the occlusal plane and the midline palatal suture. (**c**) Finally, the pre-treatment tooth of interest was superimposed to the post-treatment tooth and thus the exact positional change was recorded.

Following each superimposition of T0 and T1 models, the pre-selected teeth of interest at T0 were superimposed individually to the respective teeth at T1. This way the positional changes of each tooth crown from T0 to T1 were recorded in three dimensions, with the origin of the axis positioned on each crown centroid^23^ and the axes of movement parallel to the midline palatal suture (Y: green, anteroposterior movement; positive: anterior) and on (X: red, lateral movement; positive: right) or vertical (Z: blue, vertical movement; positive: up/apical) to the occlusal plane (Fig. 2). Rotation of each tooth around the X (red; torque, positive: buccal crown), Y (green; tip, positive: left), and Z (blue; rotation, positive: right buccal) axis was also recorded (Fig. 2).

### Accuracy, precision, and reproducibility

Initially, one researcher performed all superimpositions. The same and another calibrated researcher repeated the whole procedure 2 weeks later, independently, using the basic set of settings, to test intra- and inter-observer error.

To evaluate the accuracy obtained by each superimposition technique (areas A, B, C, D, and E), we assessed the congruence of the two models in area A by measuring the mean absolute distance (MAD)^24^ of each mesh vertex of the T0 model to the T1 surface. Only the vertices within area A, the anatomically most stable area^3, 4, 6, 7, 9, 12^, were considered in the computation, irrespective of the area used for superimposition, in order to attain a valid comparison between the methods. However, the MAD was additionally computed for each reference area, to test differences between the two ICP parameter settings.

The precision of each superimposition technique in measuring tooth movement was evaluated through the assessment of positional changes of three teeth of interest (one central incisor and two second primary molars). These variables are influenced by the accuracy of each superimposition technique and represent the main clinically relevant outcome.

Reproducibility was tested through the assessment of intra- and inter-observer error on repeated measurements of variables used for accuracy and precision assessment.

### Statistical analysis

Statistical analysis was carried out by using the SPSS (v.20.0, SPSS Inc., USA), PERMANOVA^25, 26^, and PERMDISP^27^ software.

Raw data were tested for normality through the Kolmogorov-Smirnov and Shapiro-Wilk tests and did not have a normal distribution in certain cases. Thus, non-parametric statistics were applied.

Differences in the measured variables were evaluated using permutational multivariate analysis of covariance (MANCOVA), with factorial fixed or mixed effects models. Patient was set as a covariate in all cases to account for possible matching and clustering effects. Pair-wise a posteriori comparisons were performed when significant differences were detected by the multivariate model.

Differences in accuracy between the two sets of settings were assessed by testing two crossed factors and their possible interactions: superimposition technique (fixed factor; 5 techniques) and setting (fixed factor; 2 sets of settings). The distance between models in the most stable area (A) was the variable tested. Differences in the distance of each reference area with the two settings were pairwise tested using the Wilcoxon signed-rank test. For precision testing, three crossed factors and their possible interactions were analyzed: superimposition technique (fixed factor; 5 techniques), tooth (random factor; 3 teeth), and setting (fixed factor; 2 sets of settings). All vectors of positional change of each tooth were considered as dependent variables (6 vectors: x-lateral movement, y- anteroposterior movement, z-vertical movement, x-torque, y-tip, z-rotation).

To test reproducibility, differences in accuracy and precision using the basic set of settings were assessed in the same manner. Operator (fixed factor; 2 operators) or time (fixed factor; 2 time points) were the factors used instead of setting in each case.

Permutational MANCOVA was done on Euclidean distances calculated from raw data. The P-value was calculated on raw data through permutation of residuals under a reduced model, with 9999 random permutations. In cases when there were few unique permutations possible Monte Carlo asymptotic p-value was used instead^25^ (PERMANOVA).

Permutational analysis of multivariate dispersions (PERMDISP) was used to determine whether potential differences between any pair of groups were due to location, dispersion or a combination of the above.

In all cases, a two-sided significance test was carried out at an alpha level of 0.05. Bonferroni correction was applied for pairwise a posteriori multiple comparison tests.

To overcome potential limitations of methods described above^10^, the Bland-Altman method (difference plot)^11^ was also used to evaluate agreement between the gold standard technique (A) and all other techniques in the assessment of tooth movement.

### Data availability

The datasets generated during and/or analyzed during the current study are available from the corresponding author on reasonable request.

## Results

The main effects of different operators and time points on accuracy measurements were not significant, implying sufficient intra- and inter-operator agreement. The effect of different settings was also non-significant. Furthermore, no significant interaction was detected in any of the above tests (Table 1).

**Table 1:** Non parametric MANCOVA on accuracy measurements (deviation between structures) by different operators, time points, and settings.

Operator factor	d.f.	F	P
Covariate (Patient)	1	0.018	0.889
Superimposition	4	7.982	0.000*
Operator	1	2.400	0.125
Superimposition x Operator	4	0.808	0.530
Residual	149		
Total	159		
**Comparison** ^**1**^	**p – Operator 1**		**p – Operator 2**
A vs. B	0.010**		0.010**
A vs. C	0.000**		0.006**
A vs. D	0.000**		0.001**
A vs. E	0.003**		0.001**
B vs. C	0.605		0.959
B vs. D	0.002**		0.003**
B vs. E	0.007**		0.000**
C vs. D	0.001**		0.000**
C vs. E	0.006**		0.000**
D vs. E	0.918		0.010**
**Time factor**	**d.f**.	**F**	**p**
Covariate (Patient)	1	0.000	0.988
Superimposition	4	10.509	0.000*
Time	1	0.858	0.348
Superimposition x Time	4	1.547	0.198
Residual	149		
Total	159		
**Comparison** ^**1**^	**p – Time 1**		**p – Time 2**
A vs. B	0.010**		0.000**
A vs. C	0.000**		0.001**
A vs. D	0.000**		0.000**
A vs. E	0.003**		0.000**
B vs. C	0.605		0.836
B vs. D	0.002**		0.002**
B vs. E	0.007**		0.000**
C vs. D	0.001**		0.000**
C vs. E	0.006**		0.000**
D vs. E	0.918		0.030
**Setting factor**	**d.f**.	**F**	**p**
Covariate (Patient)	1	0.018	0.889
Superimposition	4	6.395	0.000*
Setting	1	0.006	0.937
Superimposition x Setting	4	0.066	0.992
Residual	149		
Total	159		
**Comparison** ^**1**^	**p – Setting 1**		**p – Setting 2**
A vs. B	0.010**		0.001**
A vs. C	0.000**		0.000**
A vs. D	0.000**		0.000**
A vs. E	0.003**		0.001**
B vs. C	0.605		0.469
B vs. D	0.002**		0.001**
B vs. E	0.007**		0.001**
C vs. D	0.001**		0.001**
C vs. E	0.006**		0.002**
D vs. E	0.918		0.501

Two crossed factors and their interactions were analyzed in each case having “patient” as a covariate: superimposition technique (fixed factor; 5 techniques) and operator (fixed factor; 2 operators) or time (fixed factor; 2 time points) or setting (fixed factor; 2 settings).

*p < 0.05.

^1^Pair-wise *a posteriori* tests between superimposition techniques using the *Wilcoxon signed-rank test*.

**p < 0.01; Bonferroni correction applied.

A, B, C, D, E correspond to the five superimposition techniques and reference areas tested in the study.

Different superimposition techniques showed significant differences in accuracy in all cases (Table 1). Permutational analysis of multivariate dispersions showed that differences were not only due to location (Table 2), but also due to dispersion (operator: d.f. = 4, F = 3.40, p = 0.012; time: d.f. = 4, F = 6.08, p = 0.000; setting: d.f. = 4, F = 5.24, p = 0.000). Pair-wise *a posteriori* tests showed that in most cases all techniques differed from each other (p < 0.01), except for the B and C and in certain cases D and E superimpositions (Table 1). Technique A was the most accurate, as expected, followed by B and C superimpositions that presented similar level of accuracy. D superimposition was consistently less accurate than A, B, and C superimpositions. E superimposition was the least accurate, showing larger differences from other techniques (Table 2).

**Table 2:** Accuracy values of each superimposition technique.

	A	B	C	D	E
Operator 1 – Setting 1 – M1	0.077 (0.05)	0.085 (0.07)	0.093 (0.07)	0.114 (0.09)	0.141 (0.11)
Operator 1 – Setting 1 – M2	0.078 (0.05)	0.095 (0.07)	0.085 (0.06)	0.116 (0.08)	0.211 (0.15)
Operator 1 – Setting 2 – M1	0.075 (0.05)	0.085 (0.06)	0.088 (0.07)	0.112 (0.08)	0.138 (0.14)
Operator 2 – Setting 1 – M1	0.082 (0.05)	0.105 (0.08)	0.092 (0.07)	0.115 (0.11)	0.178 (0.08)

Values represent median (interquartile range) of mean absolute distance (MAD) between corresponding form-stable structures (area A) in millimeters (n = 16 patients).

A, B, C, D, E correspond to the five superimposition techniques and reference areas tested in the study.

M: Measurement.

When tested pairwise, 98% estimated overlap of meshes (setting 2) resulted in most cases in slightly smaller MAD values between corresponding reference areas, but difference to 100% estimated overlap of meshes (setting 1) was negligible (Table 3).

**Table 3:** Congruence of reference areas in the two different settings.

	A	B	C	D	E
Operator 1 – Setting 1	0.077 (0.05)	0.084 (0.08)	0.093 (0.05)	0.163 (0.09)	0.060 (0.03)
Operator 1 – Setting 2	0.075 (0.05)	0.082 (0.08)	0.092 (0.04)	0.159 (0.09)	0.062 (0.03)
p-value*	0.001*	0.001*	0.001*	0.001*	0.918

Values represent median (interquartile range) of mean absolute distance (MAD) between corresponding reference areas (A, B, C, D, or E) used each time in millimeters (n = 16 patients).

*p < 0.01, Wilcoxon signed-rank test, Bonferroni correction applied.

A, B, C, D, E correspond to the five superimposition techniques and reference areas tested in the study.

Although the main effects of operator and time on precision measurements were statistically significant (Table 4), the precision of all techniques was high, since the magnitude of these effects was negligible (less than 0.5 mm and 1° in all cases, except technique E) (Supplementary Tables S1 and S2). The effect of setting on precision measurements was not significant (Table 4). In all cases, the detected tooth movement by each superimposition technique differed significantly (Table 4). Permutational analysis of multivariate dispersions showed that differences between techniques were partially due to dispersion (operator: d.f. = 3, F = 415.5, p = 0.028; time: d.f. = 3, F = 261.7, p = 0.009; setting: d.f. = 3, F = 45.8, p = 0.000). A significant interaction of operator and precision of each superimposition technique was detected (Table 4). Further investigation revealed that differences between operators tended to increase when the superimposition reference area was smaller (Supplementary Table S1). A similar effect was also evident for repeated measurements (Table 4 and Supplementary Table S2) and different settings (Table 4 and Supplementary Table S3).

**Table 4:** Non parametric MANCOVA on precision measurements (tooth movement) performed by different operators, time points, and settings.

Operator factor	d.f.	F	p
Covariate (Patient)	1	5.816	0.002*
Superimposition	4	38.771	0.000*
Tooth	2	21.277	0.000*
Operator	1	24.204	0.001*^1^
Superimposition x Tooth	8	0.048	1.000
Superimposition x Operator	4	21.140	0.000*
Tooth x Operator	2	0.009	1.000
Superimposition x Tooth x Operator	8	0.006	1.000
Residual	449		
Total	479		
**Comparison** ^**2**^	**p**		
A vs. B	0.000*^1^		
A vs. C	0.000*^1^		
A vs. D	0.000*^1^		
A vs. E	0.001*^1^		
B vs. C	0.000*^1^		
B vs. D	0.001*^1^		
B vs. E	0.002*^1^		
C vs. D	0.000*^1^		
C vs. E	0.000*^1^		
D vs. E	0.000*^1^		
**Time factor**	**d.f**.	**F**	**p**
Covariate (Patient)	1	4.232	0.010*
Superimposition	4	20.349	0.000*
Tooth	2	21.704	0.000*
Time	1	16.847	0.003*^1^
Superimposition x Tooth	8	0.079	1.000
Superimposition x Time	4	4.488	0.001*
Tooth x Time	2	0.026	1.000
Superimposition x Tooth x Time	8	0.032	1.000
Residual	449		
Total	479		
**Comparison** ^**2**^	**p**		
A vs. B	0.003*^1^		
A vs. C	0.004*^1^		
A vs. D	0.001*^1^		
A vs. E	0.002*^1^		
B vs. C	0.000*^1^		
B vs. D	0.102^1^		
B vs. E	0.015*^1^		
C vs. D	0.001*^1^		
C vs. E	0.001*^1^		
D vs. E	0.003*^1^		
**Setting factor**	**d.f**.	**F**	**p**
Covariate (Patient)	1	5.432	0.003*
Superimposition	4	27.412	0.000*
Tooth	2	22.987	0.000*
Setting	1	1.035	0.438^1^
Superimposition x Tooth	8	0.069	1.000
Superimposition x Setting	4	4.106	0.001*
Tooth x Setting	2	0.038	1.000
Superimposition x Tooth x Setting	8	0.009	1.000
Residual	449		
Total	479		
**Comparison** ^**2**^	**p**		
A vs. B	0.001*^1^		
A vs. C	0.000*^1^		
A vs. D	0.001*^1^		
A vs. E	0.002*^1^		
B vs. C	0.000*^1^		
B vs. D	0.006*^1^		
B vs. E	0.003*^1^		
C vs. D	0.000*^1^		
C vs. E	0.001*^1^		
D vs. E	0.005*^1^		

Three crossed factors and their possible interactions were analyzed in each case having patient as a covariate: superimposition technique (fixed factor; 5 techniques), tooth (random factor; 3 teeth), and operator (fixed factor; 2 operators) or time (fixed factor; 2 time points) or setting (fixed factor; 2 settings). All vectors of positional change of each tooth were considered as dependent variables (6 vectors: x-lateral movement, y-anteroposterior movement, z-vertical movement, x-torque, y-tip, z-rotation).

*p < 0.05.

^1^Monte Carlo asymptotic p-value.

^2^Tests among levels of the factor Superimposition.

A, B, C, D, E correspond to the five superimposition techniques and reference areas tested in the study.

**Figure 3:**
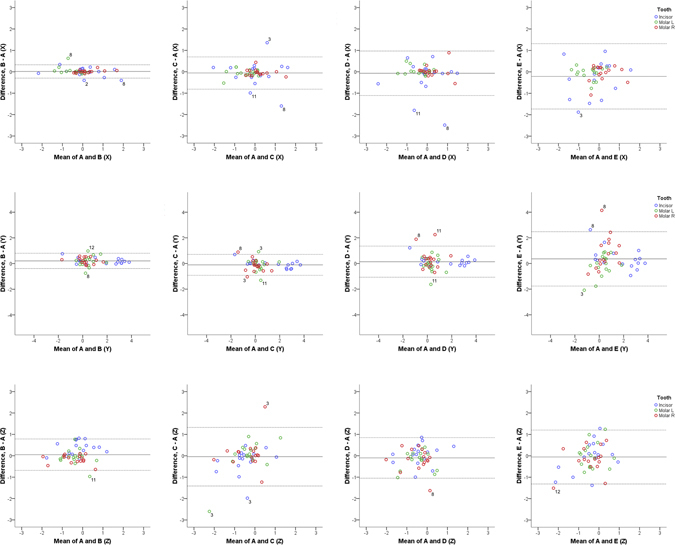
Differences of B, C, D, and E from the gold standard technique (distances). Bland Altman plots of differences of B, C, D and E superimposition techniques from the gold standard superimposition technique regarding the measured tooth movements of the three teeth of interest in the three planes of space (mm). The axes length represents the true range of observed values of structural changes. The continuous horizontal line shows the mean and the dashed lines the 95% confidence intervals. Point labels represent patients with values located outside the 95% confidence intervals of each set of measurements.

**Figure 4:**
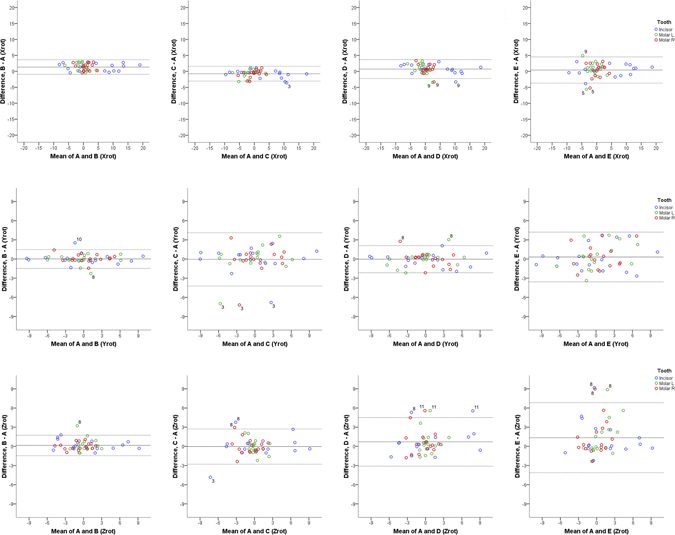
Differences of B, C, D, and E from the gold standard technique (rotations). Bland Altman plots of differences of B, C, D and E superimposition techniques from the gold standard superimposition technique regarding the measured tooth rotations of the three teeth of interest in the three planes of space (°). The axes length represents the true range of observed values of structural changes. The continuous horizontal line shows the mean and the dashed lines the 95% confidence intervals. Point labels represent patients with values located outside the 95% confidence intervals of each set of measurements.

In all cases, tooth factor, had a significant effect on precision measurements (Table 4), as expected due to the special features of the tested teeth (anterior tooth actively involved and posterior teeth not involved in treatment). However, no interaction of tooth type with any other factor, including superimposition technique, was detected. This is also evident in the Bland-Altman plots (Figs 3 and 4).

One-sample t-test showed that mean differences of each superimposition technique (B, C, D, and E) from the gold standard (A) technique in detected tooth movements (precision) were significantly different from 0 only in four out of 24 cases tested (p < 0.003; Bonferroni correction applied) and all were of negligible magnitude. These regarded the rotation of teeth around the x-axis, detected through superimpositions B, C, and D and the movement of teeth on the y-axis detected through superimposition B (Supplementary Table S4). However, Bland-Altman plots revealed individual differences of the B, C, D, and E from the gold standard (A) superimposition. Technique B showed more similar results to the gold standard technique in contrast to techniques C, D, and E which showed larger differences (Figs 3 and 4, and Supplementary Table S4). There was no evidence that the extent of difference between techniques was related to the extent or the direction of tooth movement in either case (Figs 3 and 4).

**Figure 5:**
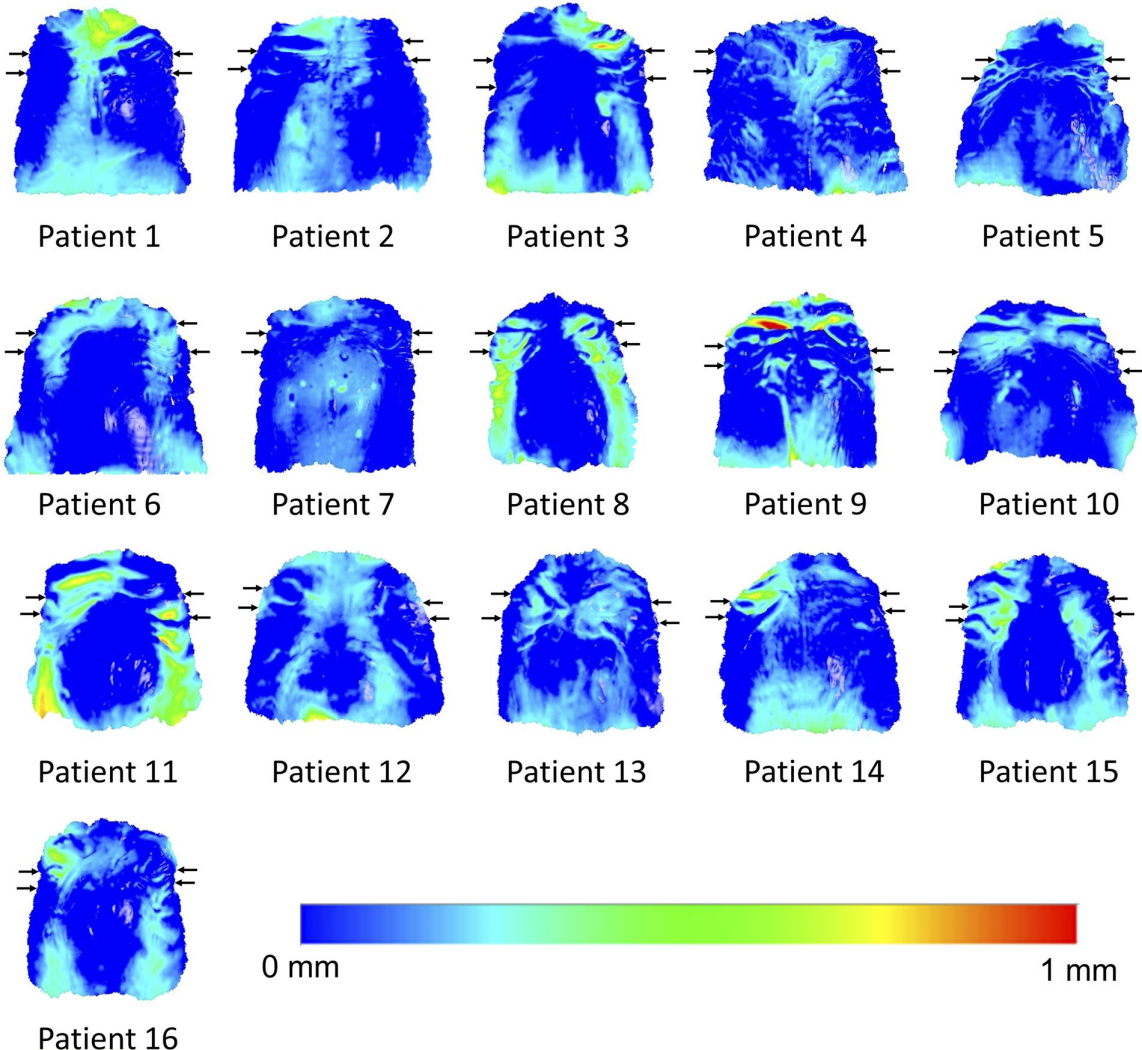
Color maps showing morphological differences in the palate between T0 and T1. Superimposition of T0 and T1 models of each patient in area D. The extent of congruence between serial models in each case is shown with specific color coding. In each case, the upper pair of black arrows shows the position of the second rugae and the lower pair the third rugae. Note that patients 3, 8, and 11 show reduced congruence of the superimposed models on regions that have been used as superimposition references. Patient 9 shows also reduced congruence, but on a region that was not used as a reference.

The higher differences mainly concerned three specific patients, implying reduced superimposition technique performance in these cases (Figs 3 and 4). After testing the differences in whole palate morphology between T0 and T1, it became evident that these three patients had considerable morphological differences in parts of the superimposition reference areas (Patient 3, 8, and 11; Fig. 5).

## Discussion

The present report is the first to thoroughly test the effect of reference area on superimposition of serial 3D digital dental models. Indeed, the reference area had a considerable influence on superimposition outcome, despite the fact that all reference areas, except from area E, shared the common area of the medial part of the third rugae and the small area dorsal to it. Furthermore, this is the first study that tested the palatal superimposition outcome on patients with active growth on the palate. At this developmental stage, mainly targeted preventive or interceptive treatments, such as the one in our sample, are provided to patients, and thus, our results are expected to be applicable to most cases of this stage and also to growth studies of untreated subjects.

The Mean Absolute Distance (MAD) was the metric chosen to assess the distance between corresponding surface areas in superimposed datasets. In contrast to Root Mean Squared Distance (RMSD), which weighs large distances more, this metric gives the same weight to all distances^24^. Thus, potentially large distances due to bubbles in the impression or similar local artifacts would not bias the findings.

In the present study, pre-existing regular dental casts obtained from a private practice were used to test the performance of different superimposition techniques in an everyday clinical setting. Thus, the quality of the casts was average, allowing for true representation of artifacts that occur in everyday practice. Such examples could be the red areas on the first ruga region of Patient 9 and on the left second ruga of Patient 3 that are shown in Fig. 5. When increased distances between T0 and T1 models were evident in the reference areas, this led to higher differences in measured tooth movement by different superimposition techniques, as was the case for Patients 3, 8, and 11, shown in Fig. 5. Differences in palatal morphology of Patient 8 could also be due to growth, since they were symmetrical throughout the whole palate. In any case, the results of the present study were not affected by these inconsistencies, as seen in the Bland-Altman plots (Figs 3 and 4) and are, thus, applicable to casts obtained in everyday clinical practice from similar patient groups.

The Bland Altman difference plots facilitated the assessment of superimposition techniques in each individual patient and allowed for the detailed investigation of the performance of each technique in the sample. Since mean differences of each technique from the gold standard (A) were not significantly different from 0, this further supported the validity of the gold standard technique. However, the findings suggested that techniques C, D, and E should be avoided due to larger differences from the gold standard technique in individual cases, while technique B showed better performance. These results highlight the importance of the evaluation of differences between techniques in individual cases and the inadequacy of mean comparison methods for the evaluation of such hypotheses, as previously suggested^10, 11^.

Although the overall effect of setting factor on accuracy was not significant, when tested pairwise, 98% estimated overlap of meshes resulted in most cases in smaller MAD values between corresponding reference areas. A possible reason for this could be the robustness of the superimposition using 98% overlap to local deformations of the reference areas caused by impression or cast formation flaws. Nevertheless, the magnitude of differences compared to 100% overlap was minimal. Thus, based on the present results, we suggest 100% overlap of meshes as a standard setting, which is a safer option (since it uses the whole reference area) that provides valid results also for models with regular artifacts.

The effect of different operators and time points tended to be higher when the reference area was smaller. This occurred probably because small differences in reference area selection had a bigger effect when the total reference area was relatively small. However, the magnitude of this effect was small and thus, this factor alone cannot argue in favor of the use of larger reference areas, when considering the whole set of the present results.

### Limitations

The gold standard reference used in this study was supported by the almost complete congruence of serial models in this area, but still another solid reference, such as metal markers, would be useful as an additional confirmation of the findings.

The use of regular casts that could include artifacts as those occurring in everyday practice offers a true representation of current clinical conditions, but could also account for part of the differences detected by different techniques. The tests using 98% overlap of meshes were performed to control for this factor and provided similar results.

The range of the initial patient’s age and the time lapse between serial casts could also affect results, but bivariate correlations of these factors with accuracy values were all weak and not statistically significant. Thus, even if there was such an effect that could not be detected due to power considerations, it is expected to be negligible (Supplementary Table S5).

The selection of the clinical crown of interest each time a superimposition was to be performed indeed reflects actual clinical and research conditions, but could also account for part of the differences detected by different techniques, since differences in the selected object would lead to different position of the centroid, which is the origin of the axes where tooth movement was assessed. To account for this, the operators were instructed to carefully select the whole clinical crown each time. Indeed, visual assessment of repeated selections revealed almost identical models.

## Conclusions

The present study clearly demonstrated that the reference area used for palatal superimposition of serial 3D dental models has an important influence on superimposition outcome in growing patients. The superimposition on the medial part of the third rugae and a small area dorsal to it provides accurate, reproducible, and precise results. Incorporation of a line throughout the midline palatal suture to the third rugae area provides comparable results. Incorporation of other rugae or palatal areas was not supported, as well as an area on the posterior palate, that did not include any rugae.

Further research should be performed in other patient groups, if possible with additional stable reference markers, to further confirm and generalize these findings. The establishment of methods of 3D superimposition of serial 3D dental models is of great importance for the dental community, since it provides a large amount of detailed information on alterations in structural morphology caused by treatment, growth and/or potential pathology, without subjecting the patients to risks, such as those of radiation exposure. The methodology presented in our study, can be used to facilitate these purposes both in clinical and research settings.

## Electronic supplementary material

Supplementary Information
