# Supplementary material for: Assessment of different techniques for 3D superimposition of serial digital maxillary dental casts on palatal structures
Source: Sci Rep. 2017 Jul 19;7:5838. doi: 10.1038/s41598-017-06013-5 (PMC5517608; doi:10.1038/s41598-017-06013-5)
Supplement: Supplementary file 1 — Supplementary Information [file 41598_2017_6013_MOESM1_ESM.pdf]

Assessment of different techniques for 3D superimposition of serial digital maxillary dental casts on palatal structures

Georgios Vasilakos, Roman Schilling, Demetrios Halazonetis & Nikolaos Gkantidis

Supplementary Table S1. Mean of absolute differences of tooth movement measurements performed by the two operators.

		A	B	C	D	E
**Linear measurements**						
**Incisor**	X (mm)	0.21	0.29	0.28	0.08	0.78
	Y (mm)	0.15	0.13	0.13	0.08	0.44
	Z (mm)	0.25	0.28	0.17	0.10	0.72
**Molar R**	X	0.10	0.10	0.11	0.02	0.16
	Y	0.17	0.24	0.21	0.04	0.75
	Z	0.23	0.23	0.27	0.10	0.34
**Molar L**	X	0.10	0.12	0.11	0.02	0.18
	Y	0.23	0.20	0.22	0.09	0.87
	Z	0.21	0.25	0.32	0.08	0.38
	Median	0.21	0.23	0.21	0.08	0.44
**Angular measurements**						
**Incisor**	X-rot (°)	0.65	0.73	0.55	0.37	2.25
	Y-rot (°)	0.70	0.86	0.71	0.54	0.88
	Z-rot (°)	0.45	0.74	0.62	0.28	1.98
**Molar R**	X-rot	0.51	0.57	0.30	0.21	2.29
	Y-rot	0.54	0.64	0.85	0.18	0.89
	Z-rot	0.48	0.68	0.72	0.18	1.76
**Molar L**	X-rot	0.65	0.69	0.41	0.28	2.44
	Y-rot	0.53	0.65	0.89	0.20	0.92
	Z-rot	0.65	0.71	0.63	0.37	1.97
	Median	0.54	0.69	0.63	0.28	1.97

Friedman test showed significant differences between methods ($p=0.000$).
Pair-wise *a posteriori* tests between superimposition techniques using the *Wilcoxon signed-rank test* showed that all techniques differed significantly to each other except from techniques A from C and B from C ($p<0.01$; Bonferroni correction applied).
A, B, C, D, E correspond to the five superimposition techniques and reference areas tested in the study.

Supplementary Table S2. Mean of absolute differences of tooth movement measurements performed at two different time points.

		A	B	C	D	E
**Linear measurements**						
	X (mm)	0.18	0.32	0.25	0.11	0.57
**Incisor**	Y (mm)	0.09	0.27	0.16	0.08	0.41
	Z (mm)	0.19	0.20	0.20	0.08	0.59
	X	0.32	0.11	0.05	0.02	0.16
**Molar R**	Y	0.53	0.27	0.25	0.13	0.67
	Z	0.51	0.20	0.29	0.11	0.35
	X	0.36	0.09	0.08	0.02	0.10
**Molar L**	Y	0.52	0.22	0.21	0.08	0.78
	Z	0.58	0.22	0.32	0.08	0.31
	Median	0.36	0.22	0.21	0.08	0.41
**Angular measurements**						
	X-rot (°)	0.58	1.22	0.82	0.47	1.60
**Incisor**	Y-rot (°)	0.78	1.68	0.84	0.54	0.90
	Z-rot (°)	0.53	0.69	0.68	0.30	1.72
	X-rot	0.97	0.70	0.48	0.34	1.80
**Molar R**	Y-rot	0.90	0.53	0.63	0.17	0.85
	Z-rot	1.03	0.73	0.71	0.34	1.48
	X-rot	0.95	0.57	0.58	0.40	1.73
**Molar L**	Y-rot	1.13	0.50	0.93	0.22	0.91
	Z-rot	1.18	0.76	0.77	0.53	1.70
	Median	0.95	0.70	0.71	0.34	1.6

Friedman test showed significant differences between methods ($p=0.000$).
Pair-wise *a posteriori* tests between superimposition techniques using the *Wilcoxon signed-rank test* showed that all techniques differed significantly to each other except from techniques A from B and B from C ($p<0.01$; Bonferroni correction applied).
A, B, C, D, E correspond to the five superimposition techniques and reference areas tested in the study.

Supplementary Table S3. Mean of absolute differences of tooth movement measurements performed with two different settings.

		A	B	C	D	E
**Linear measurements**						
**Incisor**	X (mm)	0.13	0.17	0.17	0.11	0.40
	Y (mm)	0.09	0.05	0.09	0.05	0.32
	Z (mm)	0.14	0.12	0.11	0.07	0.27
**Molar R**	X	0.08	0.11	0.06	0.86	0.10
	Y	0.10	0.11	0.17	0.71	0.45
	Z	0.08	0.07	0.12	0.99	0.44
**Molar L**	X	0.05	0.04	0.05	0.03	0.15
	Y	0.10	0.13	0.12	0.11	0.34
	Z	0.09	0.08	0.13	0.05	0.36
	Median	0.09	0.11	0.12	0.11	0.34
**Angular measurements**						
**Incisor**	X-rot (°)	0.59	0.55	0.62	0.33	1.05
	Y-rot (°)	0.84	0.79	0.75	0.53	1.03
	Z-rot (°)	0.44	0.62	0.51	0.50	1.52
**Molar R**	X-rot	0.34	0.21	0.40	0.25	0.74
	Y-rot	0.20	0.40	0.35	0.17	1.13
	Z-rot	0.47	0.56	0.54	0.58	1.19
**Molar L**	X-rot	0.35	0.23	0.33	0.28	0.90
	Y-rot	0.29	0.38	0.44	0.29	1.21
	Z-rot	0.39	0.49	0.41	0.32	1.18
	Median	0.39	0.49	0.44	0.32	1.13

Friedman test showed significant differences between methods ($p=0.000$).
Pair-wise *a posteriori* tests between superimposition techniques using the *Wilcoxon signed-rank test* showed that all techniques differed significantly to each other except from techniques A from B, A from D, B from C, B from D, and C from D ($p<0.01$; Bonferroni correction applied).
A, B, C, D, E correspond to the five superimposition techniques and reference areas tested in the study.

Supplementary Table S4. Differences between each superimposition technique from the "gold standard" technique considering the detected tooth movements. One sample t-test shows if the mean difference is significantly different from zero in each case.

						95% Confidence Interval	
		**t**	**df**	**P**	**Mean Difference**	**Lower**	**Upper**
	B - A	0.663	47	0.511	0.015	-0.031	0.061
**X** (mm)	C - A	-1.065	47	0.292	-0.059	-0.171	0.053
	D - A	-0.886	47	0.380	-0.068	-0.221	0.086
	E - A	-1.882	47	0.066	-0.211	-0.437	0.015
	B - A	4.705	47	0.000*	0.207	0.118	0.296
**Y** (mm)	C - A	-1.760	47	0.085	-0.106	-0.228	0.015
	D - A	1.546	47	0.129	0.138	-0.042	0.318
	E - A	2.303	47	0.026	0.359	0.045	0.672
	B - A	0.922	47	0.361	0.050	-0.059	0.158
**Z** (mm)	C - A	-0.430	47	0.669	-0.043	-0.246	0.159
	D - A	-1.425	47	0.161	-0.100	-0.241	0.041
	E - A	-0.616	47	0.541	-0.057	-0.244	0.129
	B - A	8.069	47	0.000*	1.351	1.014	1.688
**X – rot** (°)	C - A	-4.245	47	0.000*	-0.723	-1.066	-0.380
	D - A	3.453	47	0.001*	0.753	0.314	1.191
	E - A	1.445	47	0.155	0.437	-0.171	1.045
	B - A	0.052	47	0.959	0.006	-0.211	0.223
**Y – rot** (°)	C - A	-0.170	47	0.866	-0.052	-0.670	0.565
	D - A	-0.118	47	0.906	-0.019	-0.333	0.296
	E - A	1.098	47	0.278	0.315	-0.262	0.892
	B - A	1.301	47	0.200	0.153	-0.084	0.390
**Z – rot** (°)	C - A	-0.178	47	0.859	-0.036	-0.443	0.370
	D - A	2.495	47	0.016	0.699	0.135	1.263
	E - A	3.350	47	0.002	1.353	0.540	2.166

*p<0.002; Bonfrerroni correction applied
A, B, C, D, E correspond to the five superimposition techniques and reference areas tested in the study.

Supplementary Table S5. Bivariate correlations (Spearman's rho, 2-tailed) of patient's age at treatment start and time lapse (T0 to T1) between serial dental models with accuracy values.

	**A**		**B**		**C**		**D**		**E**	
	**rho**	**p**	**rho**	**p**	**rho**	**p**	**rho**	**p**	**rho**	**p**
**Operator 1 – Setting 1 – M1**										
Patient age	0.02	0.931	-0.04	0.888	0.01	0.957	0.01	0.966	0.06	0.829
T0-T1 time	0.22	0.418	0.27	0.311	0.16	0.542	0.39	0.134	0.26	0.322
**Operator 1 – Setting 1 – M2**										
Patient age	0.13	0.617	0.11	0.688	0.03	0.914	0.06	0.837	0.10	0.721
T0-T1 time	0.25	0.350	0.28	0.284	0.18	0.513	0.38	0.140	0.40	0.125
**Operator 1 – Setting 2 – M1**										
Patient age	0.02	0.931	-0.03	0.897	0.06	0.829	0.02	0.931	-0.06	0.837
T0-T1 time	0.23	0.399	0.31	0.240	0.17	0.528	0.35	0.188	0.21	0.431
**Operator 2 – Setting 1 – M1**										
Patient age	0.20	0.458	0.11	0.688	0.11	0.680	0.14	0.610	-0.22	0.412
T0-T1 time	0.16	0.557	0.33	0.204	0.20	0.458	0.33	0.204	0.17	0.520

$p<0.05$
A, B, C, D, E correspond to the five superimposition techniques and reference areas tested in the study.
M: Measurement
